# Comparative Analysis of Atezolizumab Plus Bevacizumab and Hepatic Artery Infusion Chemotherapy in Unresectable Hepatocellular Carcinoma: A Multicenter, Propensity Score Study

**DOI:** 10.3390/cancers15174233

**Published:** 2023-08-24

**Authors:** Ji Hoon Kim, Hee-Chul Nam, Chang-Wook Kim, Hee Sun Cho, Jae-Sung Yoo, Ji Won Han, Jeong Won Jang, Jong Young Choi, Seung Kew Yoon, Hyun Yang, Si Hyun Bae, Suho Kim, Jung Suk Oh, Ho Jong Chun, Chang Ho Jeon, Jaegyoon Ahn, Pil Soo Sung

**Affiliations:** 1 Department of Gastroenterology and Hepatology, Uijeongbu St. Mary’s Hospital, The Catholic University of Korea, Seoul 06591, Republic of Korea; jihoon23@gmail.com (J.H.K.); hcnam128@catholic.ac.kr (H.-C.N.); cwkim@catholic.ac.kr (C.-W.K.); 2 Department of Gastroenterology and Hepatology, Seoul St. Mary’s Hospital, The Catholic University of Korea, Seoul 06591, Republic of Korea; jhs-cho@hanmail.net (H.S.C.); imjsyoo@gmail.com (J.-S.Y.); tmznjf@catholic.ac.kr (J.W.H.); garden@catholic.ac.kr (J.W.J.); jychoi@catholic.ac.kr (J.Y.C.); yoonsk@catholic.ac.kr (S.K.Y.); 3 Department of Gastroenterology and Hepatology, Eunpyeong St. Mary’s Hospital, The Catholic University of Korea, Seoul 06591, Republic of Korea; oneggu@naver.com (H.Y.); baesh@catholic.ac.kr (S.H.B.); 4 Department of Radiology, Seoul St. Mary’s Hospital, The Catholic University of Korea, Seoul 06591, Republic of Korea; lucidnature@naver.com (S.K.); oj-cumc@hanmail.net (J.S.O.); chunray@catholic.ac.kr (H.J.C.); 5 Department of Radiology, Eunpyeong St. Mary’s Hospital, The Catholic University of Korea, Seoul 06591, Republic of Korea; changho.jeon@gmail.com; 6 Department of Computer Science & Engineering, Incheon National University, Incheon 22573, Republic of Korea

**Keywords:** atezolizumab plus bevacizumab, hepatic artery infusion chemotherapy, hepatocellular carcinoma, overall survival, progression-free survival

## Abstract

**Simple Summary:**

We aimed to compare the prognosis of patients with advanced hepatocellular carcinoma treated with the first-line atezolizumab plus bevacizumab (AB) combination chemotherapy and the less popular locoregional treatment mainly used in East Asia, hepatic artery infusion chemotherapy (HAIC). We conducted a retrospective study with 114 patients treated with AB and 193 patients treated with HAIC and compared the overall survival (OS) and progression-free survival (PFS). Our results showed comparable OS between the two therapy groups; however, PFS was superior in patients treated with AB combination therapy. After compensating for confounding variables via propensity score matching, there was no significant difference in PFS and OS between the two groups.

**Abstract:**

This study aimed to compare the prognosis and characteristics of patients with advanced hepatocellular carcinoma treated with first-line atezolizumab plus bevacizumab (AB) combination therapy and hepatic artery infusion chemotherapy (HAIC). We retrospectively assessed 193 and 114 patients treated with HAIC and AB combination therapy, respectively, between January 2018 and May 2023. The progression-free survival (PFS) of patients treated with AB combination therapy was significantly superior to that of patients treated with HAIC (*p* < 0.05), but there was no significant difference in overall survival (OS). After propensity score matching, our data revealed no significant differences in OS and PFS between patients who received AB combination therapy and those who received HAIC therapy (*p* = 0.5617 and 0.3522, respectively). In conclusion, our propensity score study reveals no significant differences in OS and PFS between patients treated with AB combination therapy and those treated with HAIC.

## 1. Introduction

Hepatocellular carcinoma (HCC) accounts for the largest proportion of primary liver cancers. It is one of the leading causes of cancer-related deaths worldwide [1]. Between 2000 and 2014, the incidence rates of liver cancer increased by 2.6% in men and by 3.0% in women [2]. Although the overall cancer mortality rate has decreased since the 1990s, the liver cancer mortality rate increased by 43% from 2000 to 2016 [3]. Hepatitis B virus infection is the most common cause of HCC, followed by hepatitis C virus infection, alcohol consumption, and non-alcoholic fatty liver disease (NAFLD) [4]. Recent advancements in medical technologies, including vaccination and antiviral therapies, have increased the number of patients with NAFLD due to obesity and metabolic syndrome [5]. Consequently, there has been increased interest and studies on the detection and prevention of HCC due to NAFLD and non-alcoholic steatohepatitis and the difference between NAFLD and HCCs due to other etiologies [4].

Recently, the IMbrave150 study demonstrated that the immune checkpoint inhibitor atezolizumab plus bevacizumab (AB) was superior to sorafenib in overall survival (OS) and progression-free survival (PFS) outcomes; as a result, it has been approved as the first-line treatment for unresectable locally advanced or metastatic HCC since May 2020 [6]. Several patients have been treated with combination therapy with positive results. Multiple studies have reported comparable results between AB combination therapy and lenvatinib. Between 2022 and 2023, studies by Kim et al. and Su et al. revealed a comparative OS between patients with unresectable HCC treated with AB and lenvatinib [7,8]. Lenvatinib has comparable outcomes with AB combination therapy. In fact, in 2022, Casadei-Gardini et al. and Rimini et al. suggested comparable OS in patients with advanced HCC treated with lenvatinib compared with AB combination therapy [9,10].

Another treatment option for unresectable locally advanced HCC is hepatic artery infusion chemotherapy (HAIC), which is commonly used in East Asia [11]. Approximately 8% of the patients initially diagnosed with HCC are treated with HAIC [12]. It delivers high concentrations of chemotherapy agents directly to the liver, thus decreasing systemic toxicity and increasing delivery of the agent to malignant intrahepatic lesions [13]. In 2019, Sung et al. demonstrated that intrahepatic tumor reduction elicited by HAIC prolonged the survival of patients with unresectable HCC, regardless of portal vein invasion or extrahepatic metastasis [14]. In 2021, Lee et al. suggested comparable OS and PFS between patients treated with lenvatinib and HAIC [15]. In 2020, Ueshima et al. demonstrated that in patients with macrovascular invasion without extrahepatic metastasis, HAIC had superior OS compared to sorafenib [16]. Before AB combination therapy was recognized as the first-line chemotherapy regimen for advanced HCC, Hatooka et al. suggested that HAIC might be superior to sorafenib as a first-line treatment [17]. Choi et al. demonstrated that in advanced HCC with portal vein invasion, HAIC was significantly superior to sorafenib [18]. In addition, most recently, a meta-analysis by Zhang et al. also suggested that HAIC was superior to sorafenib in advanced HCC with portal vein invasion [19]. Our institute recognized the need to compare the prognoses of patients treated with AB combination therapy and HAIC.

## 2. Patients and Methods

### 2.1. Study Population

This study was approved by the Institutional Review Board of the Catholic University of Korea (approval number: XC23TIDI0015) and was conducted in accordance with the Declaration of Helsinki. We enrolled patients diagnosed with HCC who were treated with AB combination therapy and HAIC between January 2018 and May 2023 at Seoul St. Mary’s Hospital, Eunpyeong St. Mary’s Hospital, and Euijeongbu St. Mary’s Hospital. We retrospectively reviewed the hospital records of the enrolled patients who received AB combination therapy (n = 114) and HAIC therapy (n = 193). Patients were diagnosed with HCC based on the imaging criteria of the American Association for the Study of Liver Disease and the 2022 Korean Liver Cancer Association and National Cancer Center Korea practice guidelines [20,21]. Most patients had Barcelona Clinic Liver Cancer (BCLC) stage C. Some patients had BCLC stage B but did not have indications of locoregional therapy (transarterial chemoembolization refractory or infiltrative nature).

### 2.2. Treatment Protocol

The AB combination therapy protocol consisted of 1200 mg of atezolizumab and 15 mg/kg of bevacizumab [22]. HAIC consisted of 60 mg/m^2^/day of cisplatin and 500 mg/m^2^/day of 5-fluorouracil (5-FU). Both 5-FU and cisplatin were infused on days 1–2, and only 5-FU was infused on day 3 [23]. HAIC was administered via infusion through an injection port, which was installed at the initial therapy. The port consisted of a catheter that ended at the common or proper hepatic artery and a chemoport installed in the subcutaneous pocket of the inguinal region. The port was kept throughout the treatment.

### 2.3. Endpoints and Response Evaluation

The primary endpoints were OS (time from initial treatment to death from any cause) and PFS (time from initial treatment to disease progression according to the Response Evaluation Criteria in Solid Tumors (RECIST) version 1.1, or death from any cause). The secondary endpoints were the objective response rate (ORR) (percentage of complete response (CR) or partial response (PR)) and the disease control rate (DCR) (percentage of CR or PR, or stable disease (SD)). All patients were evaluated by computed tomography or magnetic resonance imaging at diagnosis and initial treatment and were evaluated every 4–9 weeks according to the RECIST version 1.1. Adverse events were recorded according to the Common Terminology Criteria for Adverse Events, and adverse events above grade 3 were included.

### 2.4. Statistical Analyses

We calculated the OS and PFS of all enrolled patients using Kaplan–Meier analysis. To compensate for any existing confounding variables, we continued with a propensity score matching (PSM) analysis. After PSM, the OS and PFS of the matched groups were calculated and compared. All statistical analyses were performed using SPSS version 23.0 software (SPSS, Chicago, IL, USA). The Kaplan–Meier method was used for survival analyses, including OS and PFS, and differences were examined using the log-rank test. Cox regression analyses were performed to identify factors associated with survival outcomes, and factors with *p* < 0.05 in univariate analysis were included in multivariate analysis. The therapeutic efficacy was shown by the ORR and DCR using the chi-squared test. Statistical significance was defined as *p*-values < 0.05.

## 3. Results

### 3.1. Baseline Characteristics

The baseline characteristics are presented in Table 1. A total of 114 patients received AB combination therapy, and 194 patients received HAIC. There were no significant differences in age, sex, etiology of malignancy, and the Eastern Cooperative Oncology Group (ECOG) performance scores between the two groups. However, there was a significant difference in BCLC stage, Child–Pugh class, serum alpha-fetoprotein (AFP) level, tumor size, portal vein invasion, metastasis, and previous treatments between the two groups. Patients treated with AB were of a significantly more progressed BCLC stage (*p* = 0.002) than those treated with HAIC, but patients treated with AB had better Child–Pugh scores (*p* = <0.001) than those treated with HAIC. Patients treated with HAIC had significantly higher serum AFP levels and larger tumor sizes (*p* = 0.004 and *p* = 0.027, respectively) than those treated with AB. Patients treated with HAIC had significantly more portal vein invasion and less distant metastasis (*p* = 0.039 and *p* < 0.001, respectively) than those treated with AB. Our data revealed that significantly more patients treated with HAIC had previous treatment histories (*p* < 0.001).

Naturally, PSM was performed to compensate for these confounding variables. We analyzed our data using PSM with the following factors: sex, age, etiology, ECOG performance status scores, BCLC stage, Child–Pugh scores, metastasis, and tumor size (caliper = 0.2). In total, 83 pairs of patients were matched by PSM. A comparative analysis of the two groups of baseline characteristics showed no significant differences in possible confounding factors between the two groups (Table 2).

### 3.2. Treatment Responses

The treatment responses of the enrolled patients are shown in Table 3. In patients receiving AB, the median OS and PFS periods were 174.5 days and 129 days, respectively. In patients treated with HAIC, the median OS and PFS periods were 230 days and 129 days, respectively. Of the patients who received AB, 6 (5.26%) achieved CR, 26 (31.58%) PR, 43 (37.72%) SD, and 29 (25.44%) progressive disease (PD). Among the patients who received HAIC, 11 (5.70%) achieved CR, 31 (16.06%) PR, 135 (69.95%) SD, and 15 (7.77%) PD. Our statistical analysis showed a significant difference between the two groups (Table 3, *p* < 0.001). In the AB therapy group, 42 (36.84%) patients showed an objective response and 85 (74.56%) achieved disease control. In the HAIC group, 42 (21.76%) patients showed an objective response and 177 (91.71%) achieved disease control.

**Figure 1:**
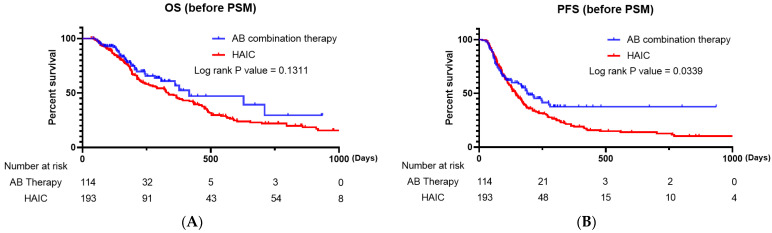
(**A**) Kaplan–Meier survival curve comparing the overall survival of patients treated with AB and HAIC (all enrolled patients). (**B**) Kaplan–Meier survival curve comparing the progression-free survival of patients treated with AB and HAIC (all enrolled patients). AB, atezolizumab plus bevacizumab; HAIC, hepatic artery infusion chemotherapy.

There was a significant difference in the ORR (*p* = 0.003) and DCR (*p* < 0.001) between the two groups. When we compared the Kaplan–Meier survival curves for PFS and OS, the AB combination therapy group had a significantly superior PFS compared to that of the HAIC group (*p* = 0.0339), and there was no significant difference in OS (*p* = 0.1311) between the two groups (Figure 1A,B).

However, when we analyzed the baseline characteristics between the two therapy groups, there were significant differences in terms of BCLC stage, Child–Pugh class, serum AFP level, tumor size, portal vein invasion, distant metastasis, and previous treatment (Table 2).

**Figure 2:**
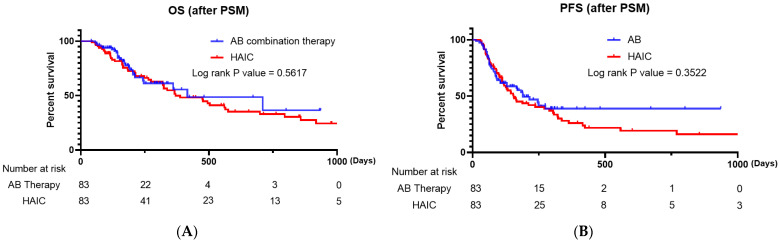
(**A**) Kaplan–Meier survival curve comparing the overall survival of patients treated with AB and HAIC (propensity-score-matched patients). (**B**) Kaplan–Meier survival curve comparing the progression-free survival of patients treated with AB and HAIC (propensity-score-matched patients). AB, atezolizumab plus bevacizumab; HAIC, hepatic artery infusion chemotherapy.

The treatment responses of matched patients via PSM are shown in Table 4. In the AB therapy group, 1 (1.20%) patient achieved CR, 29 (34.94%) PR, 33 (39.76%) SD, and 20 (24.10%) PD. In the HAIC group, 9 (10.84%) patients achieved CR, 15 (18.07%) PR, 49 (59.04%) SD, and 10 (12.05%) PD (Table 3). After PSM, a significant difference was observed between the two groups in terms of treatment response (*p* = 0.001). No significant difference was observed between the two groups regarding ORR (*p* = 0.320); however, the HAIC group had a significantly superior DCR (*p* = 0.044) (Table 3). The analysis revealed no significant differences in OS and PFS between the two groups (Figure 2B, *p* = 0.5617 and *p* = 3522, respectively).

### 3.3. Factors Associated with Survival Outcomes

We analyzed the factors associated with OS and PFS in all enrolled patients with univariate and multivariate analyses using the Cox proportional hazards model (Table 5). Factors from the univariate analysis with a *p*-value < 0.05 were included in the multivariate analysis. In the univariate analyses regarding OS, the patients’ performance, represented by ECOG performance status ≤ 1, and the patients’ liver function, represented by Child–Pugh class, were factors associated with favorable OS. In the multivariate analyses, Child–Pugh class A (hazard ratio (HR), 0.397; 95% confidence interval (CI), 0.277–0.568; *p* < 0.001) was a significant factor associated with OS. In the univariate analyses regarding PFS, Child–Pugh class A and a serum AFP level of <1000 ng/mL were factors associated with favorable PFS; distant metastasis was associated with poor PFS. In the multivariate analyses, Child–Pugh class A (HR, 0.435; 95% CI, 0.314–0.603; *p* < 0.001) was significantly associated with favorable PFS, and distant metastasis (HR, 1.572; 95% CI, 1.182–2.092; *p* = 0.002) was significantly associated with poor PFS.

### 3.4. Adverse Events

We also assessed the adverse events that occurred during treatment in both groups (Table 6). Significantly more adverse events occurred in the HAIC therapy group than in the AB combination therapy group. The most common adverse event in the HAIC therapy group was hyperbilirubinemia.

## 4. Discussion

To the best of our knowledge, no study has compared AB combination therapy and HAIC in patients with advanced HCC. This was the first real-world study that compared first-line systemic chemotherapy for unresectable HCC with the less popular locoregional chemotherapy, HAIC. Our study included PSM analysis, and 83 propensity-score-matched enrolled patients were analyzed. After PSM analysis, our results revealed no significant difference in OS and PFS between patients who received AB and those who received HAIC (*p* = 0.5617 and *p* = 0.3522, respectively).

AB therapy is the first-line chemotherapy currently used to treat patients with unresectable advanced HCC; durvalumab plus tremelimumab is currently not available in South Korea, but if the first-line treatment is not feasible, sorafenib or lenvatinib can be considered [24]. Since the IMbrave150 trial demonstrated that AB therapy was superior in OS and PFS to sorafenib chemotherapy in patients with advanced HCC, several studies regarding AB therapy have been conducted. Cheon et al. confirmed the efficacy and safety of AB therapy in Korean patients and noted inferior outcomes in patients with an elevated neutrophil-to-lymphocyte ratio [25]. Fulgenzi et al. also confirmed the safety and efficacy of AB therapy and pointed out that the presence of portal vein invasion and a higher albumin–bilirubin grade were correlated with a poor prognosis [26]. Recently, Casadei-Gardini et al. suggested that there was no significant difference in OS between AB therapy and lenvatinib [27]. Interestingly, Persano et al. reported higher ORR in patients treated with lenvatinib compared with those treated with AB therapy [28]. D’Alessio et al. published a notable study confirming the safety of AB combination therapy in patients with liver functions of Child–Pugh classes A and B [29].

Unlike other common locoregional treatments, including transarterial chemoembolization or transarterial radioembolization, which is used more commonly [30], HAIC is a locoregional chemotherapy technique that does not involve embolization; thus, it can be considered a type of systemic chemotherapy directly infused into the liver [11,31,32,33]. In 2014, Song et al. revealed the comparative OS and time to progression between patients with advanced HCC with portal vein tumor thrombosis treated with sorafenib and HAIC [34]. Considering these results, our institute acknowledged the need to compare the prognoses of patients with unresectable HCC treated with AB therapy and HAIC.

There may be questions regarding the difference between the results before and after PSM analysis. Before propensity matching, there were significant differences between patients treated with HAIC and AB, including the BCLC stage, Child–Pugh scores, serum AFP level, tumor size, portal vein invasion, and distant metastasis. More patients treated with HAIC had BCLC stages A and B. AFP levels and tumor size were significantly higher in patients treated with HAIC than in those treated with AB, which may be because several terminal patients were palliatively treated with HAIC despite worse liver function. Portal vein invasion was more common in patients treated with HAIC than in those treated with AB, possibly because HAIC is preferred more so in patients with portal vein invasion [34]. Distant metastasis occurred in a significantly larger proportion of patients in the AB therapy group than in the HAIC therapy group, as systemic chemotherapy is preferred over locoregional therapy in patients with distant metastasis. To compensate for confounding variables, we performed PSM analyses. In total, 83 matches were selected, and the results were compared. After PSM analysis, no significant difference was observed between the two therapy groups. Considering that AB combination therapy has been confirmed as the first-line chemotherapy for patients with advanced HCC because of its safety and efficacy [35], this result may be further validated by future prospective studies.

The limitations of this study include its retrospective design. Several enrolled patients were previously treated with other therapies before being treated with AB therapy or HAIC; therefore, confounding variables may have affected our data and results, and temporal relationships may have been missed. Because of resource restrictions, we could not analyze the temporal relationships of treatments known to have immunomodulation effects, including transarterial chemoembolization and radiotherapy, with AB combination and HAIC therapies [36,37].

## 5. Conclusions

Before PSM, AB combination therapy appeared superior to HAIC therapy in terms of PFS but comparable in OS. However, after PSM, AB combination and HAIC therapies seemed to have similar efficacy. A prospective cohort study with a meticulous design can help elucidate accurate differences between current first-line systemic chemotherapy, AB therapy, and HAIC.

## Figures and Tables

**Table 1:** Baseline characteristics of enrolled patients (whole data).

	AB Combination Therapy (n = 114)	HAIC (n = 193)	*p*-Value
Age, y	63.28 (11.86)	61.99 (11.75)	0.658
Sex			0750
Male	99 (86.84%)	170 (88.08%)	
Female	15 (13.16%)	23 (11.92%)	
BCLC stage			0.002
0/A (very early, early)	0	2 (1.1%)	
B (intermediate)	9 (9.7%)	44 (23.5%)	
C (advanced)	105 (90.3%)	147 (75.4%)	
D (end stage)	0	0	
Etiology			0.893
HBV	73 (64.04%)	127 (65.80%)	
HCV	4 (3.51%)	6 (3.11%)	
Alcohol use	20 (17.54%)	28 (14.51%)	
Others	17 (14.91%)	32 (16.58%)	
Child–Pugh class			<0.001
A	106 (92.98%)	135 (69.95%)	
B	8 (7.02%)	55 (28.50%)	
C	0	3 (1.55%)	
ECOG performance status score			0.060
0	83 (72.81%)	150 (77.72%)	
1	28 (24.56%)	30 (15.54%)	
2	3 (2.63%)	13 (6.74%)	
3	0	0	
4	0	0	
Serum AFP level (ng/mL)	9621.65 (19,063.01)	15,280.12 (34,030.43)	0.004
Tumor size	6.51 (5.66)	8.43 (4.81)	0.027
Portal vein invasion			0.039
No	55 (48.25%)	59 (30.57%)	
Yes	59 (51.75%)	123 (63.73%)	
Distant metastasis			<0.001
No	46 (40.35%)	145 (75.13%)	
Yes	68 (59.65%)	48 (24.87%)	
Previous treatment			<0.001
No	69 (60.53%)	76 (39.38%)	
Yes	45 (39.47%)	117 (60.62%)	

Data are presented as n (%) and means ± standard deviations. AB, atezolizumab plus bevacizumab; HAIC, hepatic artery infusion chemotherapy; BCLC stage, Barcelona Clinic Liver Cancer stage; HBV, hepatitis B virus; HCV, hepatitis C virus; ECOG performance status, Eastern Cooperative Oncology Group performance status; AFP, alpha-fetoprotein.

**Table 2:** Baseline characteristics of enrolled patients (after propensity score matching).

	AB Combination Therapy (n = 83)	HAIC (n = 83)	*p*-Value
Age, y	62.64 (12.55)	61.92 (13.00)	0.978
Sex			0.633
Male	72 (86.75%)	74 (89.16%)	
Female	11 (13.25)	9 (10.84)	
BCLC stage			
0/A (very early, early)	0	0	0.417
B (intermediate)	9 (10.84%)	6 (7.23%)	
C (advanced)	74 (89.16%)	77 (92.77%)	
D (end stage)	0	0	
Etiology			0.533
HBV	50 (60.24%)	55 (66.27%)	
HCV	3 (3.61%)	3 (3.61%)	
Alcohol use	17 (20.48%)	10 (12.08%)	
Others	13 (15.66%)	15 (18.07%)	
Child–Pugh class			1.000
A	75 (90.36%)	75 (90.36%)	
B	8 (9.64%)	8 (9.64%)	
C	0	0	
ECOG performance status score			0.984
0	60 (72.29%)	61 (73.49%)	
1	21 (25.30%)	20 (24.10%)	
2	2 (2.41%)	2 (2.41%)	
3	0	0	
4	0	0	
Serum AFP level (ng/mL)	10,746.41 (19,713.23)	16,524.46 (36,228.35)	0.204
Tumor size	8.21 (5.49)	8.18 (5.24)	0.969
Portal vein invasion			0.098
No	32 (38.55%)	22 (26.51%)	
Yes	51 (61.45%)	61 (73.49%)	
Distant metastasis			0.347
No	44 (53.01%)	50 (60.24%)	
Yes	39 (46.99%)	33 (39.76%)	
Previous treatment			0.349
No	40 (48.19%)	34 (40.96%)	
Yes	43 (51.81%)	49 (59.04%)	

Data are presented as n (%) and means ± standard deviations. AB, atezolizumab plus bevacizumab; HAIC, hepatic artery infusion chemotherapy; BCLC stage, Barcelona Clinic Liver Cancer stage; HBV, hepatitis B virus; HCV, hepatitis C virus; ECOG performance status, Eastern Cooperative Oncology Group performance status; AFP, alpha-fetoprotein.

**Table 3:** Treatment responses in enrolled patients (whole data).

Treatment Responses	AB Combination Therapy (n = 114)	HAIC (n = 193)	*p*-Value
			<0.001
CR	6 (5.26%)	11 (5.70%)	
PR	36 (31.58%)	31 (16.06%)	
SD	43 (37.72%)	135 (69.95%)	
PD	29 (25.44%)	15 (7.77%)	
ORR	42/114 (36.84%)	42/193 (21.76%)	0.003
DCR	85/114 (74.56%)	177/193	<0.001

AB, atezolizumab plus bevacizumab; HAIC, hepatic artery infusion chemotherapy; CR, complete response; PR, partial response; SD, stable disease; PD, progressive disease; ORR, objective response rate; DCR, disease control rate.

**Table 4:** Treatment responses in enrolled patients (after propensity score matching).

Treatment Responses	AB Combination Therapy (n = 83)	HAIC (n = 83)	*p*-Value
			0.001
CR	1 (1.20%)	9 (10.84%)	
PR	29 (34.94%)	15 (18.07%)	
SD	33 (39.76%)	49 (59.04%)	
PD	20 (24.10%)	10 (12.05%)	
ORR	30/83 (36.14%)	24/83 (28.92%)	0.320
DCR	63/83 (75.90%)	73/83 (87.95%)	0.044

AB, atezolizumab plus bevacizumab; HAIC, hepatic artery infusion chemotherapy; CR, complete response; PR, partial response; SD, stable disease; PD, progressive disease; ORR, objective response rate; DCR, disease control rate.

**Table 5:** Univariate and multivariate analyses of factors associated with OS and PFS in the entire cohort.

Variables	Overall Survival			Progression-Free Survival		
	Univariate (*p*-Value)	Multivariate (*p*-Value)	HR (95% CI)	Univariate (*p*-Value)	Multivariate (*p*-Value)	HR (95% CI)
AB therapy vs. HAIC	0.132			0.034		
Age	0.306			0.226		
Sex	0.735			0.743		
Etiology	0.052			0.446		
Tumor size	0.150			0.100		
Serum AFP < 1000 ng/mL	0.111			0.046	0.131	0.810 (0.616–1.065)
ECOG performance status 0 and 1	0.002	0.078	0.929 (0.738–2.435)	0.151		
Distant metastasis	0.071			0.005	0.002	1.572 (1.182–2.092)
Portal vein invasion	0.964			0.517		
Child–Pugh class A	<0.001	<0.001	0.397 (0.277–0.568)	<0.001	<0.001	0.435 (0.314–0.603)
Previous treatment	0.132			0.061		

AB, atezolizumab plus bevacizumab; HAIC, hepatic artery infusion chemotherapy; HR, hazard ratio; CI, confidence interval; ECOG performance status, Eastern Cooperative Oncology Group performance status; AFP, alpha-fetoprotein.

**Table 6:** Adverse events (above grade 3) in enrolled patients.

Adverse Events	AB Combination Therapy (n = 114)	HAIC (n = 193)	*p*-Value
Total	22	69	0.001
AST/ALT (>×5 ULN)	8	32	0.016
Colitis	3	3	0.510
Fatigue	3	8	0.013
Pneumonitis	1	0	0.192
Hyperbilirubinemia	2	26	0.001
Skin rash	1	0	0.192
Anaphylactic shock	1	0	0.192
Myositis	1	0	0.192
Asthma	1	0	0.192
Thyroiditis	1	0	0.192

Data are presented as n (%) and means ± standard deviations. AB, atezolizumab plus bevacizumab; HAIC, hepatic artery infusion chemotherapy; AST, aspartate aminotransferase; ALT, alanine aminotransferase.

## Data Availability

The data presented in this study are available upon request from the corresponding author.
